# Parametric Design Optimisation of Proximal Humerus Plates Based on Finite Element Method

**DOI:** 10.1007/s10439-018-02160-6

**Published:** 2018-11-01

**Authors:** Ali Jabran, Chris Peach, Zhenmin Zou, Lei Ren

**Affiliations:** 1 School of Mechanical, Aerospace and Civil Engineering, University of Manchester, Sackville Street, Manchester, M13 9PL UK; 2 Department of Shoulder and Elbow Surgery, University Hospital of South Manchester, Southmoor Road, Wythenshawe, Manchester, M23 9LT UK

**Keywords:** Proximal humerus fractures, Finite element method, Parametric design, Constrained optimisation

## Abstract

Optimal treatment of proximal humerus fractures remains controversial. Locking plates offer theoretical advantages but are associated with complications in the clinic. This study aimed to perform parametric design optimisation of proximal humerus plates to enhance their mechanical performance. A finite element (FE) model was developed that simulated a two-part proximal humerus fracture that had been treated with a Spatial Subchondral Support (S3) plate and subjected to varus bending. The FE model was validated against *in vitro* biomechanical test results. The predicted load required to apply 5 mm cantilever varus bending was only 0.728% lower. The FE model was then used to conduct a parametric optimisation study to determine the orientations of inferomedial plate screws that would yield minimum fracture gap change (i.e. optimal stability). The feasible design space was automatically identified by imposing clinically relevant constraints, and the creation process of each FE model for the design optimisation was automated. Consequently, 538 FE models were generated, from which the obtained optimal model had 4.686% lower fracture gap change (0.156 mm) than that of the manufacturer’s standard plate. Whereas its screws were oriented towards the inferomedial region and within the range of neck-shaft angle of a healthy subject. The methodology presented in this study promises future applications in patient-specific design optimisation of implants for other regions of the human body.

## Introduction

Among the most common fractures of the human body are those of the proximal humerus. While the majority of these fractures can be treated conservatively, surgical intervention is required for complex cases.^24^ Plate-based Open Reduction Internal Fixation (ORIF) has increased in popularity as a treatment modality in recent years, owing partially to the development of locking screw technology. *In vitro* mechanical studies reveal that proximal humerus fractures treated with locking plates exhibit higher stiffness and load at failure than those with non-locking plates.^38^,^40^ However, clinical studies report high rates of postoperative complications, especially the humeral head varus collapse, glenohumeral joint penetration of screws and sub-acromial impingement of plate.^13^,^26^,^33^ Modern locking plates employ several design features to help minimise the risk of these complications. A prime example is that of their screws that are directed towards the inferomedial region of humerus, a region critical for humeral head’s stability against varus collapse.^19^,^48^

A common approach for improving implant design is by systematically conducting a set of *in vitro* biomechanical tests on potential plate designs where design selection is based on a trial and error process, which is very time consuming and resource expensive. Modern advances in computational power and algorithms have allowed performance of techniques such as the finite element (FE) analysis for implant design optimisation. This *in silico* approach allows changing of individual design parameters in isolation and testing without the issue of environmental or inter-specimen variations. It also allows parallel testing of multiple designs to reduce the cost, time and resource limitations that are often associated with *in vitro* and *in vivo* testing.

FE studies on implant design can be categorised as either biomechanical comparisons or parametric optimisation studies. Early FE studies belong to the first category as they were biomechanical comparisons of different existing plates and screw configurations used in the clinic.^8^ For example, Cegonino *et al*. investigated the functional performance of femur after distal femur fracture and fixation by different types of implants.^8^ Such comparative studies are still performed today as demonstrated by Zhou *et al*., who compared the biomechanical stability and stress of two locking compression plates for middle femoral fracture.^49^ Many FE studies on proximal humerus plates belong to this category as they investigated the effect of bone cement augmentation, medial support and screw configurations.^15^,^23^,^44^,^47^

The second category of FE studies involves optimisation of implant design by parametrising its design features (e.g. implant length). Kayabasi *et al*. used FE analysis for systemical selection of geometrical parameters to minimise the peak stress of a hip prosthesis.^29^ Similarly, Er *et al*. developed an FE model of an ankle treated with four different types of syndesmotic screws.^14^ FE analyses were conducted to find the optimal combination of screw parameters using Taguchi’s robust design method.^16^ Wee *et al*. developed a set of FE models of a diaphyseal midshaft fracture treated with a plate.^41^ Surrogate mathematical models were developed using multivariate regression to understand the relationships between different design parameters and implant performances (maximum stress in plate and screws, axial and shear strain, and axial, torsional and bending stiffness). Willing and Kim conducted parametric design optimisation of a total knee replacement where abrasive wear of the polyethylene insert was minimised under an ISO standard test for total knee replacement wear.^43^

Although numerous FE-based comparison implant studies are found in the literature, FE-based parametric optimisation studies are significantly fewer and those of proximal humerus plates are non-existent. This scarcity may be due to the fact that parametric optimisation places additional requirements on FE modelling. First, the FE model must be an accurate representation of the scenario being simulated (*in vitro* or *in vivo*). This is critical as it ultimately determines the validity of the optimised design. Second, the preparation and analysis time of the FE model must be short enough, especially when a high number of designs are to be tested in a given time-frame. These two requirements often contradict each other and specialised knowledge and experience is needed to simplify the FE model so that they are fast to analyse and yet sufficiently accurate. For example, Willing and Kim kept the mesh element count low enough for successful completion of analysis.^43^ Likewise, Hsu performed parametric optimisation of spine interbody fusion device to improve their subsidence resistance and this required simplification of loading conditions and geometry.^27^ Finally, the FE model must be robust enough to allow changes to its design parameters during optimisation. For large-scale optimisation studies, the FE model preparation eventually requires automation, ideally one which is error-free and demands minimum human intervention.

Considering these requirements, we devised this parametric optimisation study, using proximal humerus plates as an example. The aim of this study was to perform parametric optimisation design of the Spatial Subchondral Support (S3) proximal humerus plate to enhance the bone-plate construct’s varus bending stability. We developed an FE model of a proximally fractured humerus that had been implanted with S3 plate and subjected to varus bending. This model was validated with data from *in vitro* mechanical tests and used to conduct a parametric optimisation study to find optimal orientations of plate’s inferomedial screws that maximise varus bending stability of bone-plate construct. Although similar methodologies have been proposed in the literature, this study is novel in two aspects.^21^,^43^ First, it involves automation of significant parts of the FE modelling procedure. Second, it includes a constraint implementation procedure before the FE design optimisation to filter out FE models that do not meet the clinical requirements. Together, these innovative approaches reduce the preparation time of the FE models whist preserving its accuracy, and will be a step towards the development of patient-specific implants.^22^

## Materials and Methods

### FE Model Development

An FE model simulating the *in vitro* varus bending of S3 plate (Zimmer Biomet, IN, USA) was developed. To achieve this, a synthetic left humerus specimen (model 1028; Pacific Research Laboratories, Vashon, WA, USA), was scanned using a Computed Tomography (CT) scanner (SOMATOM, Siemens, Munich, Germany). An 83 mm long S3 plate for the left humerus was scanned using a FaroArm laser scanner (Faro Technologies, Lake Mary, FL, USA). The slice images were segmented using Mimics 16.0 software (Materialise, Leuven, Belgium). Surface geometries of both the plate and the bone were processed using Geomagic Wrap 2014 (3D Systems, Rock Hill, SC, USA), and were then converted into 3D solid models. To simulate the two-part fracture, the humerus was cut off at 210 mm away from the head apex, and the section of the bone between 50 and 60 mm from the head apex was also removed. Screws were modelled as cylinders and merged to the plate to produce a single part.^23^,^30^

**Figure 1:**
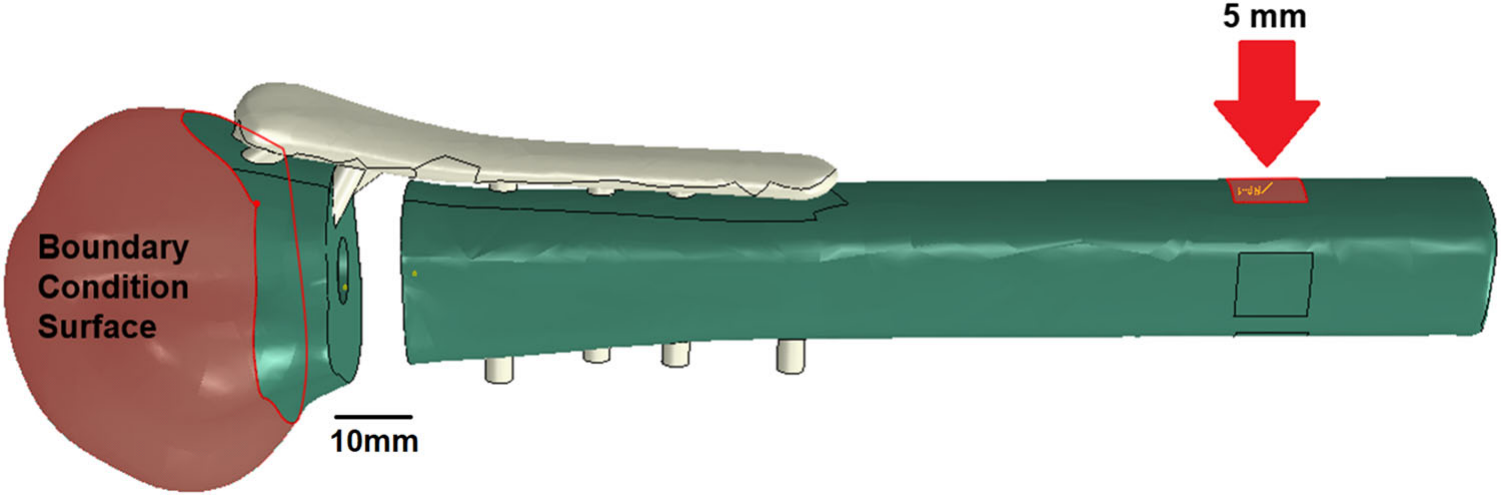
Assembly of humerus and plate in the FE model and selection of the head boundary condition surface and the shaft surface to apply varus displacement (red arrow).

To construct the FE model, solid models of humeral head, shaft and the plate were imported as 3D deformable parts into Abaqus CAE Standard 6.13 (Dassault Systemes Simulia Corp, Providence, RI, USA) and assembled according to the manufacturer’s guidelines.^4^ All three models were assigned a linear elastic isotropic material. Based on the manufacturers’ specifications, S3 plate was modelled as stainless steel 316L and the humerus from solid rigid polyurethane (Table 1).^1^,^3^,^37^ The humeral head was fixed rigidly by applying an encastre boundary condition to the section of the humeral head up to 40 mm away from the humeral head apex (Fig. 1). The humeral shaft was connected to the humeral head* via* S3 plate. A square surface with 10 mm side length was created on the humeral shaft, facing the sagittal plane and located 180 mm distal to the humeral head apex. This surface was to be displaced in order to produce varus bending. To do so, it was coupled to a reference point and a 5-mm displacement was applied to the shaft in the varus direction* via* this point. The plate and its screws were modelled as a single part to represent the ideal locking mechanism. All screws were tied to their corresponding screw holes* via* tie constraint with surface-to-surface discretisation method where the screw surfaces were set as master and the hole surfaces as slave.^47^

**Table 1:** Material and mesh properties of the plate and the humeral head and shaft

	Young’s modulus (MPa)	Poisson’s ratio	Element count
Humeral head	176.38	0.3	276,998
Humeral shaft	176.38	0.3	205,104
S3 plate	193,000	0.3	26,409

All parts were meshed using a 10-node quadratic tetrahedron (C3D10) element shape type with a global seed size of 1.5 and the local seed size of 1 for the slave surfaces, resulting in a total element of 508,511 for the entire assembly (Table 1). This choice of the seed size and the final element number was determined from a mesh sensitivity analysis where models with a total element number of 152,914–2,045,170 were tested. While they all converged and differed in bending forces value (*F*_5_) by only 0.33%, their time duration ranged from 20 min to 7 days on a PC computer with 5 processing cores and 20 GB RAM. Therefore, the medium mesh density, with a simulation time of approximately of 3 h, was selected for subsequent FE models. Four additional models with Young’s modulus and Poisson’s ratio 10% greater/less than that of the medium mesh model were developed to determine the effect of material properties on the bending force (*F*_5_). A 10% change in Poisson’s ratio was found to increase *F*_5_ by 1.62–1.77%. With a 10% reduction in Young’s modulus, *F*_5_ reduced by 8.49% while a 10% increase led to a 11.85% increase in *F*_5_.

For model validation, a set of *in vitro* biomechanical tests was performed that used identical humerus and plate specimens and subjected to the same boundary condition (humeral head fixed) and loading conditions (5 mm varus displacement at the humeral shaft) as the FE model. The load required to apply the 5 mm displacement in the varus direction (*F*_5_) was then calculated using the load–displacement data obtained and compared to the *F*_5_ value obtained from the FE model.

### *In Vitro* Biomechanical Testing

Biomechanical experiments were performed on twenty synthetic humeri (model 1028; Pacific Research Laboratories, Vashon, WA, USA) that were identical to those used in FE models. Each humerus was subjected to a two-part transverse surgical neck fracture with a 10-mm fracture gap and an additional cut 210 mm distal from the humeral head apex. The fracture gap simulated the lack of medial support at the head neck junction which is a common clinical situation associated with poor clinical outcomes. While there was no gap on the lateral side as this could be closed down and reduced, there existed a functional gap since the bone communition does not contribute to construct stability. Gap’s full closure would cause significant varus rotation of the head and may lead to poor clinical outcomes.^36^

**Figure 2:**
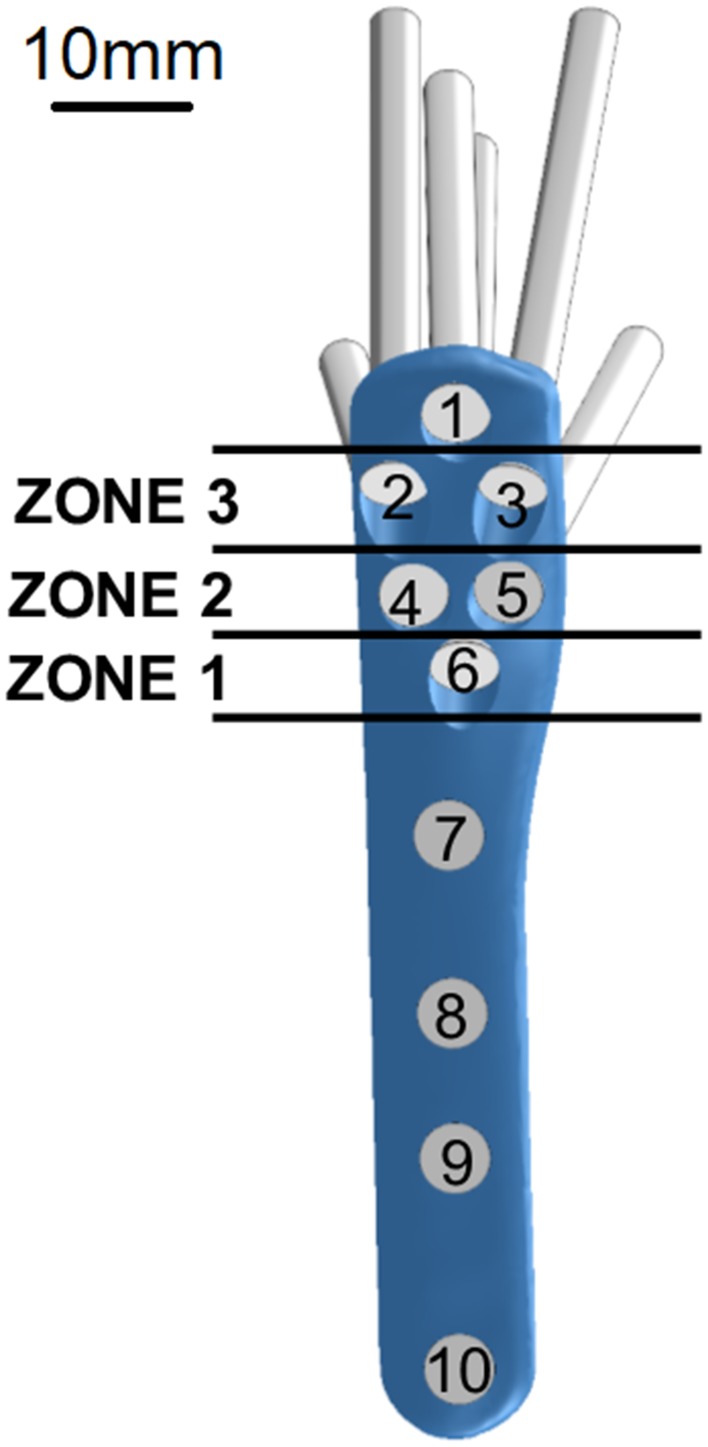
Position-based zoning of head screws of the S3 proximal humerus plate.

**Figure 3:**
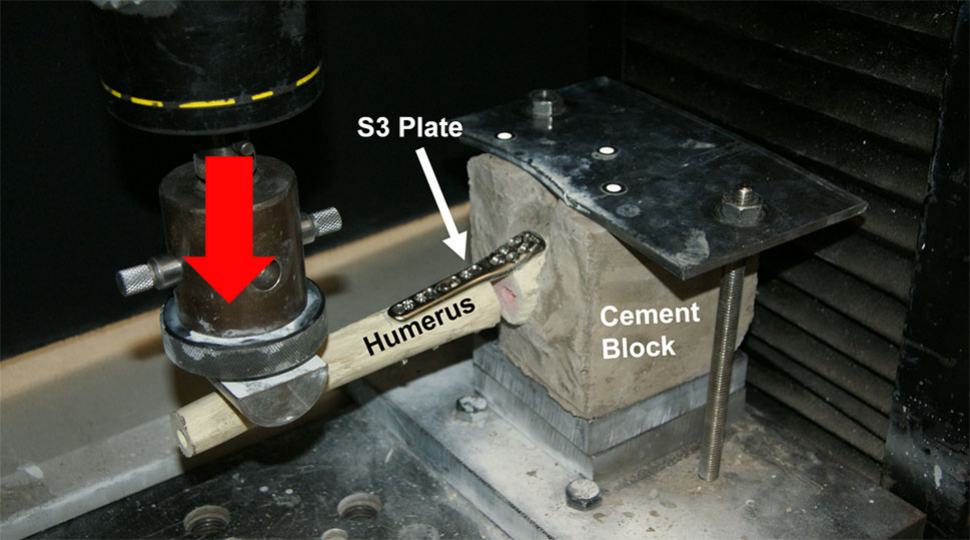
Experimental setup for varus bending tests of the S3 plate, with a load (red arrow) applied on the humeral shaft in a cantilever fashion.

For treatment, all humeri were implanted with an 83 mm long S3 plate that had four shaft and six head screw holes. Head screw holes were categorised into zones depending on their positions from the fracture site, as shown in Fig. 2. Five bone specimens were implanted with plates that had all six head screws inserted to form the control group S0. Screw length was determined in trial experiments using a Kirschner wire (Table 2). The remaining fifteen specimens were divided into three equal groups, with each group missing either zone 1, 2 or 3 screws and were appropriately labelled as configuration groups S1, S2 and S3. Humeral head was fixed with a custom-made cement block holder and clamped to a material testing machine (Instron 4500, Canton, MA, USA), in a direction perpendicular to the actuator. This setup is similar to that used by Huff *et al*.^28^ A 5 mm displacement was applied to the humeral shaft at a distance of 120 mm from the fracture site, in the varus direction at a displacement rate of 5 mm/s (Fig. 3). Based on trial experiments, these 5-mm varus displacements induced bending moments at the fracture site that were within the 0–7.5 Nm range. This replicated supraspinatus forces acting on the construct during early stages of healing under shoulder immobilisation support and was mechanically comparable to humeral immobilisation followed by a varus force acting directly at the supraspinatus insertion site.^10^,^34^,^42^ This testing procedure was repeated five times for each specimen of the four configuration groups and the obtained load and displacement data was used to determine the peak load at 5 mm displacement (*F*_5_).

**Table 2:** Descriptions and lengths (mm) of screws implanted in the configuration groups.

Configuration group	Screw number									
	1	2	3	4	5	6	7	8	9	10
S0 (control)	45, TP	45, TP	45, TP	47.5, SP	47.5, SP	55, SP	34, ND	30, M	30, ND	30, ND
S1 (no zone 1)	45, TP	45, TP	45, TP	47.5, SP	47.5, SP	None	34, ND	30, M	30, ND	30, ND
S2 (no zone 2)	45, TP	45, TP	45, TP	None	None	55, SP	34, ND	30, M	30, ND	30, ND
S3 (no zone 3)	45, TP	None	None	47.5, SP	47.5, SP	55, SP	34, ND	30, M	30, ND	30, ND

‘TP’, ‘SP’, ‘ND’ and ‘M’ refer to threaded peg, smooth peg, 90° screw and multidirectional screw respectively, while ‘None’ represents the vacant screw holes

Statistical analysis was performed in the SPSS 22.0 (IBM, NY, USA) software, using a linear mixed model approach while accounting for intra- and inter-subject variability where the random effects were defined to be the specimen and trials, fixed effects were the configuration groups and dependent variable was *F*_5_. Fisher’s least significant difference (LSD) multiple comparisons based on the least-squared means was used for pairwise comparison.

The *F*_5_ value measured for the S0 configuration group was used for the validation of the FE model.

### Parametric Optimisation

**Figure 4:**
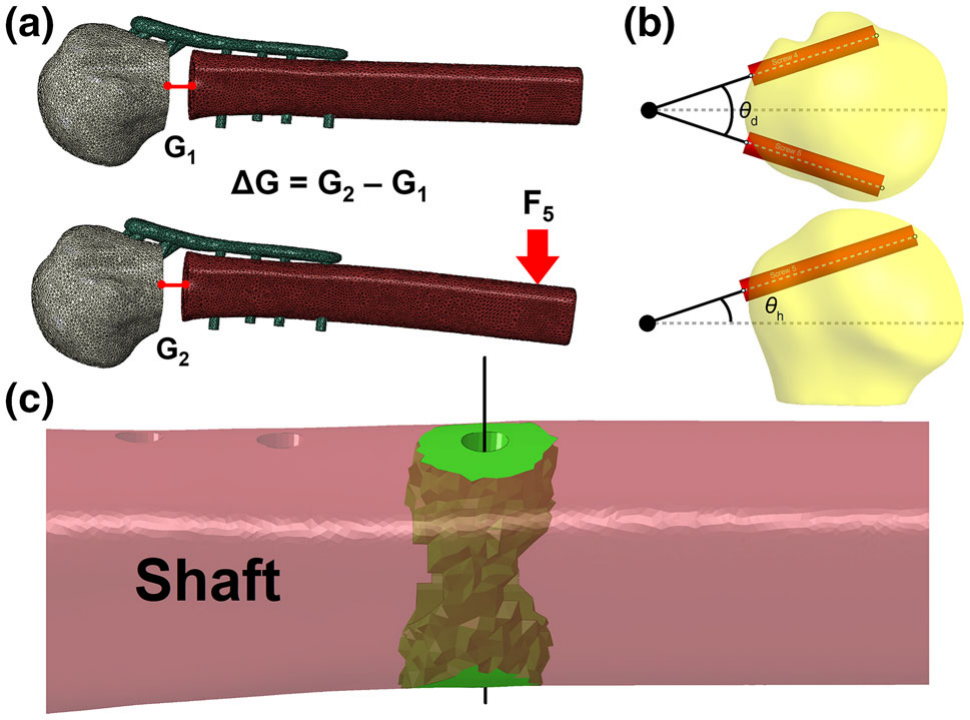
Visual representation of (a) fracture gap change calculation, (b) divergence angle *θ*_d_ and height angle *θ*_h_ of screws 4 and 5, along with screws’ midpoint (large black dot) and their midline (dashed grey line) and (c) the bone region (green) surrounding screw 10’s axis (black line) selected for stress calculations.

Out of all the zones tested, removal of zone 2 screws had the greatest effect on the construct stability (details in the Results section). This difference was statistically significant (*p* < 0.001) and made zone 2 the ideal zone for the optimisation study. The orientations of zone 2 screws (screw 4 and 5) can be described in terms of two parameters, divergence and height angle (*θ*_d_, *θ*_h_), which are the angles that the screws make with respect to their midline in the sagittal and frontal plane (Fig. 4a). In order to better quantify the stability of the bone-plate construct, the difference in the fracture gap, before and after loading, fracture gap change (Δ*G*), was proposed. This could be implemented by comparing the centroid position of the node set on either side of the fracture gap before and after loading (Fig. 4b). Thus, the primary objective of the design optimisation was to find a feasible combination of the height and divergence angles for screws 4 and 5 that yields the minimum fracture gap change in the FE analysis.

While Δ*G* minimisation can reflect the stability of the bone-plate construct, it was understood that it only reflected one aspect of the plate’s performance. This single parameter may not be able to estimate the potential risk of stress concentration on both the bone and the plate, especially at bone regions around screws. In particular, the bone region around the distal-most screw (Fig. 2, screw 10) has been reported in the literature to be a site of high stress concentration. Local stresses in this region can give more insight into the risk of further fracturing during varus bending. Thus, for each FE model in the optimisation study, the mean (*σ*_mean_) and the maximum (*σ*_max_) von Mises stress of the bone region within the 5 mm radius of screw 10 was calculated. Reduction of these stress values would indicate lower risk of failure around screw 10 (Fig. 4c).

**Figure 5:**
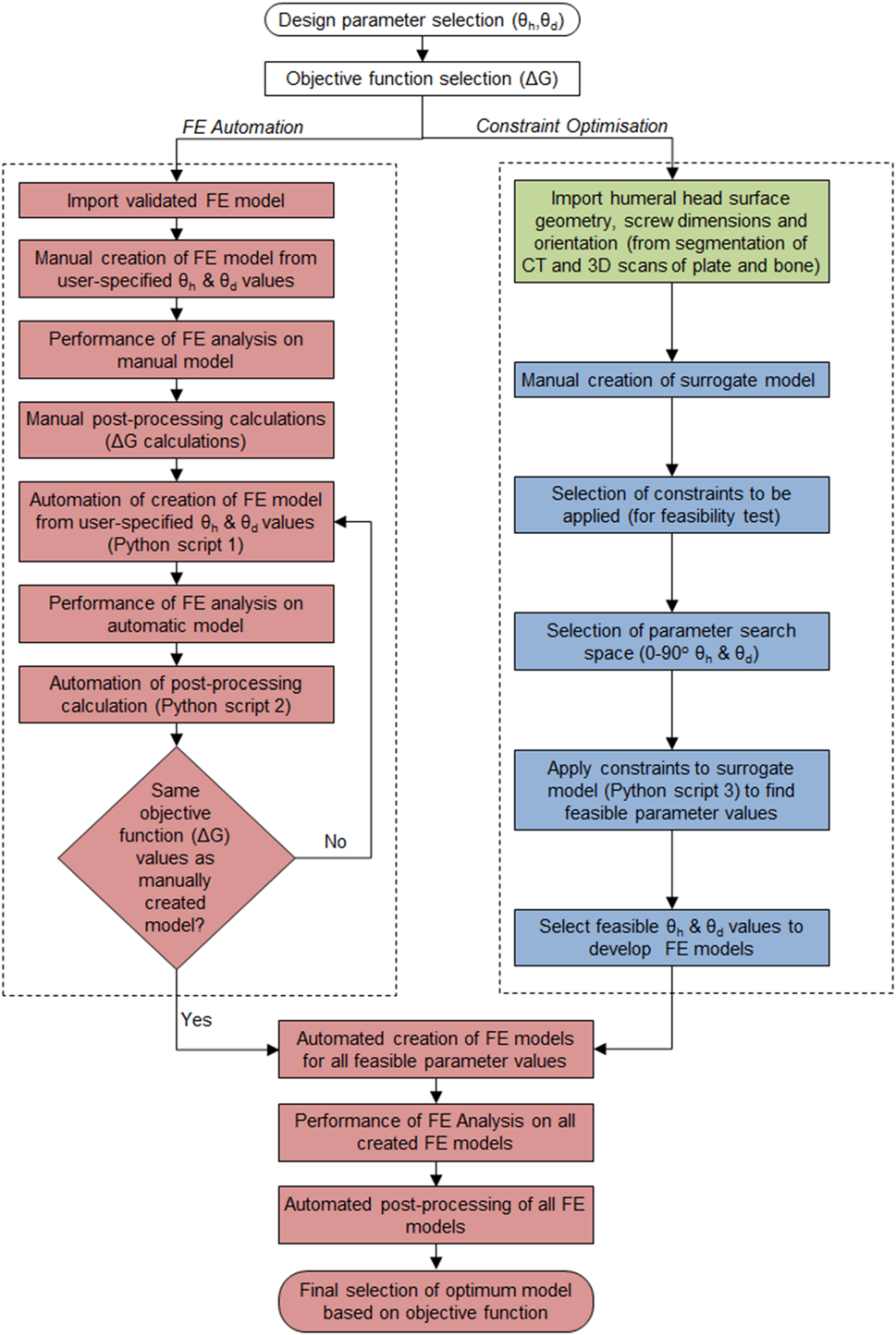
Overall workflow of the parametric optimisation study, starting from the selection of the design parameters and the object function, followed by the FE automation in Abaqus CAE (red), feasible region implementation in Geomagic Wrap (blue) and Mimics (green) and finally the creation of all 538 FE models and selection of the optimal model.

In this study, constrained optimisation was performed. The feasible region was explicitly identified using an automated procedure, which found all feasible height and divergence combinations out of a large set of candidates (Fig. 5). This would not only save computational time but also ensure that only the clinically relevant plate designs are tested. A Python script was developed and run inside the Geomagic Wrap software to apply constraints to the user-specified ranges of height and divergence angles. A range of 0°–90° was set for both height and divergence angle. This was because screws oriented at angles outside this range are clearly too far out of the humeral head. The script tested whether the screws 4 or 5 were (1) entirely outside of the humeral head, (2) in contact with the other screws’ profiles or (3) too short when in contact with the subchondral bone. If either condition was true, the height and divergence angle combination was declared unfeasible.

Identification of feasible region was implemented by developing a model that consisted of triangular surface meshes of the humeral head and six cylinders with same positions, dimensions and orientations as the head screws of S3 plate. Based on the inputted divergence and height angles, orientations of screws 4 and 5 were updated. Their maximum lengths (up to subchondral bone) were calculated by projecting their profiles onto the inner surface of the humeral head. To be considered feasible, the maximum possible length of either screw had to be at least 50 mm, with the understanding that screws ought to be long enough to reach the more distant regions of the humeral head in the treatment of complex fracture cases. Collisions among screws were tested using the line-segment to line-segment collision detection algorithm described by Lumelsky *et al*.^31^ The script was run for the full range (0°–90°) of the height and divergence angles in integer increments. The obtained feasible combinations were then used to conduct optimisation search to find optimal solution.

The identified 538 feasible height and divergence angle combinations necessitated the automation of the FE model construction. Thus, a Python script was created using Abaqus Scripting Interface, which automatically constructed the bone-plate FE model based only on the screws’ divergence and height angles. This significantly reduced the preparation time of each FE model from 8 h to only 30 s. It was used to develop a set of 538 FE models which were then submitted in batches to the supercomputer facilities at the University of Manchester to run in parallel where each job was allocated 5 cores and 20 GB RAM. An additional script was created to perform the post-processing calculation of the fracture gap based on the simulation results.

To verify if the optimal parameters (*θ*_d_ and *θ*_h_) from this set of 538 models were robust and consistent at higher and lower loading conditions, further two sets of 538 FE models were created. Models in these two sets were similar to that of the first but involved displacements of up to 2 and 10 mm, respectively. From these models, the load required to apply 2 mm (*F*_2_) and 10 mm (*F*_10_) displacements were respectively calculated, in place of *F*_5_. This was in addition to the calculation of Δ*G*, *σ*_mean_ and *σ*_max_. Further two models with 2- and 10-mm displacement were developed, both of which were based on the standard FE model.

To investigate the effect of screw length on the optimisation study’s results, an additional set of 24 FE models was developed. These were based on the most optimum design (16° divergence angle, 32° height angle) from the optimisation study in terms of Δ*G* and were subjected to 5 mm displacements. The lengths of screws 4 and 5 in these models were combinations of 0, 25 and 50, 75 and 100% of the length used in the optimisation study FE model.

## Results

### *In Vitro* Biomechanical Tests and FE Model Validation

**Figure 6:**
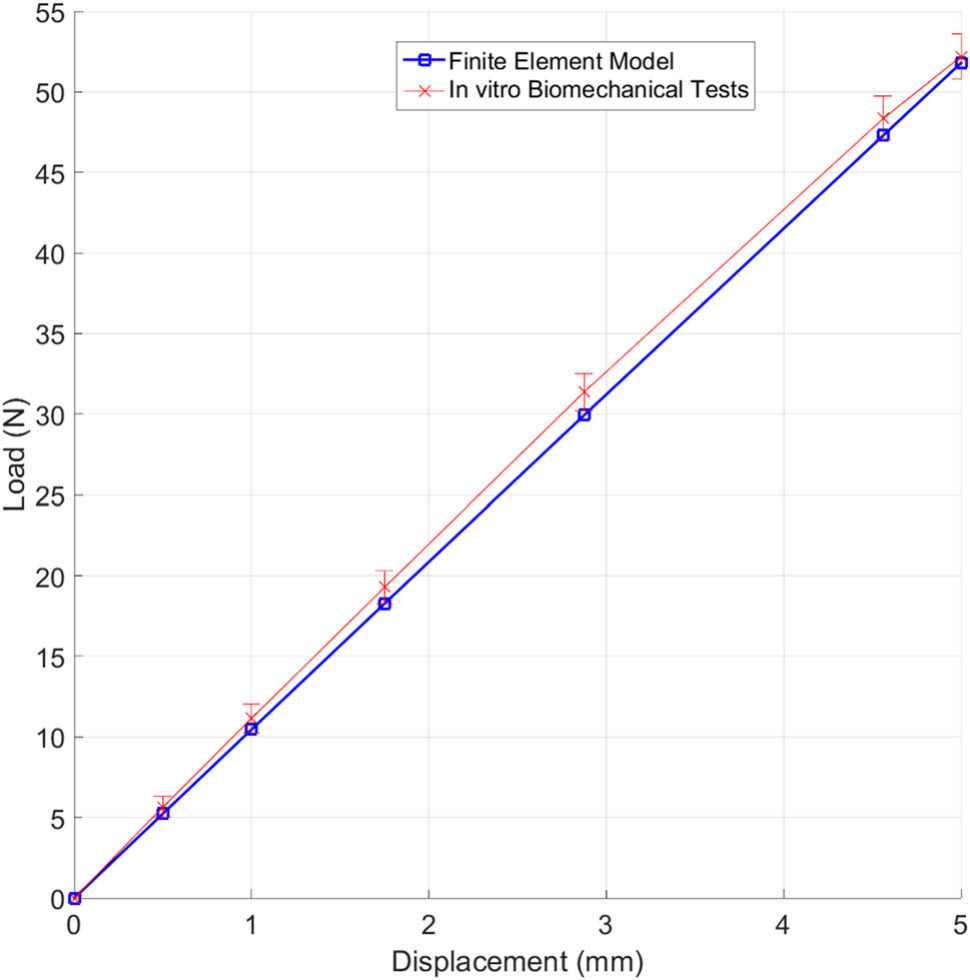
Load–displacement relationship predicted by the FE model compared with the *in vitro* biomechanical measurement data (mean ± SD).

The mean varus bending load at 5 mm displacement (*F*_5_) for the S0 group was statistically significantly higher (52.177 N ± 1.410) than S1 (41.705 N ± 1.299), followed by S3 (34.455 N ± 2.026) and S2 (32.495 N ± 0.859) configuration groups. There were statistically significant differences between all configuration pairs, with p-values less than 0.001 for all pairwise comparisons. The initial FE model simulating the standard S0 configuration group predicted a bending force (*F*_5_) of 51.797 N, which was only 0.728% lower than the 52.177 N force measured in the *in vitro* experiments. Moreover, the load–displacement curve under varus bending produced by the FE model was in very good agreement with the measurement data (Fig. 6).

**Figure 7:**
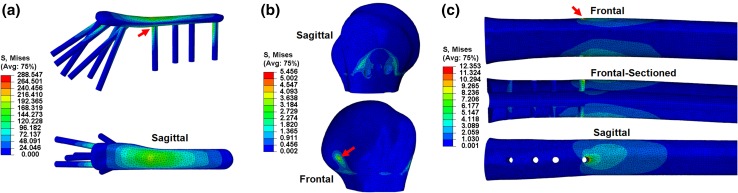
von Mises stress (MPa) distribution across the plate (a), humeral head (b) and humeral shaft (c), in the standard FE model under 5 mm varus displacement, with their respective points of maximum stress shown with red arrows.

From the three parts, the plate presented the highest value of maximum von Mises stress (288.547 MPa), followed by the humeral shaft (12.353 MPa) and the head (5.456 MPa). In the plate, high stresses were reported at the screw-plate junctions (Fig. 7a), and the maximum value was found at screw 7’s head, at the section of the plate spanning the fracture site.

On the humeral head, high stresses were noted around the insertion point of zone 3 screws, around the outlines of the fixed surface defined by the boundary condition, as well as on the back and top of the head and also around the screw holes due to the bone-screw interface (Fig. 7b).^17^ Similarly, stresses on the humeral shaft were found to be high at the screw holes, particularly at the most distal one (Fig. 7c, screw 10).

### Parametric Optimisation

**Figure 8:**
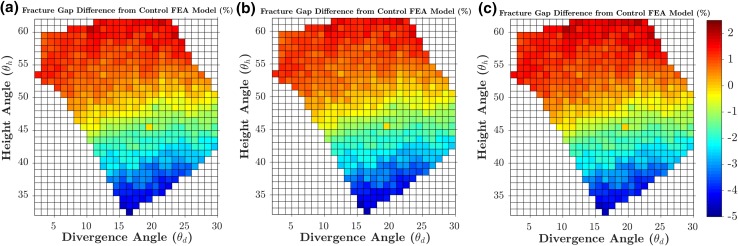
Contour plots showing the percentage changes in the fracture gap change (Δ*G*) for each of the 538 feasible height and divergence angle combinations, when subjected to 5 mm (a), 2 mm (b) and 10 mm (c) of varus displacement. Percentage changes for each loading condition are calculated with respect to the baseline values from its standard model.

**Figure 9:**
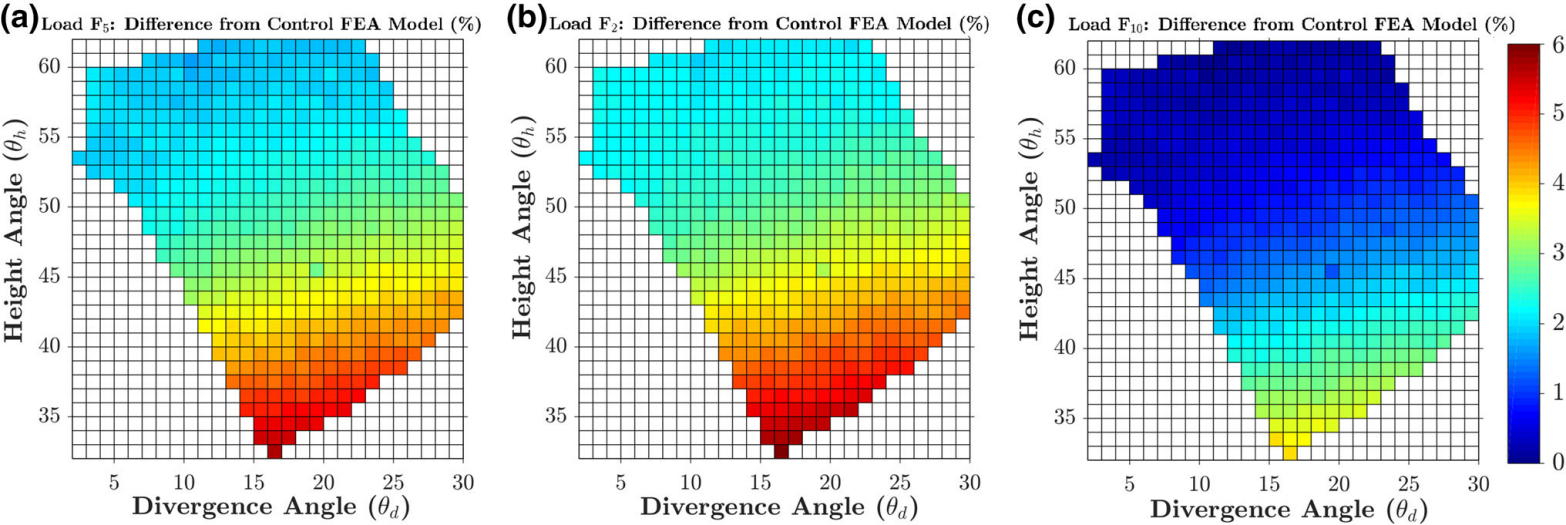
Contour plots showing the percentage changes in the peak load (*F*_5_, *F*_2_, *F*_10_) for each of the 538 feasible height and divergence angle combinations, when subjected to 5 mm (a), 2 mm (b) and 10 mm (c) of varus displacement. Percentage changes for each loading condition are calculated with respect to the baseline values from its standard model.

**Figure 10:**
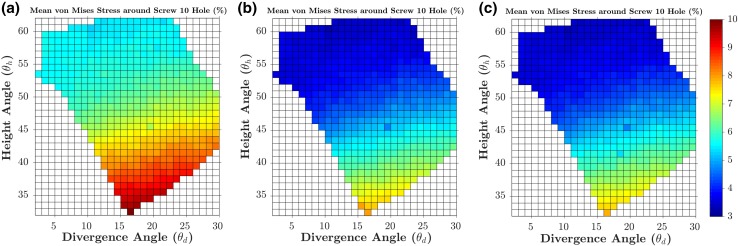
Contour plots showing the percentage changes in the mean von Mises stress in the bone region 5 mm around screw 10, for each of the 538 feasible height and divergence angle combinations, when subjected to 5 mm (a), 2 mm (b) and 10 mm (c) of varus displacement. Percentage changes for each loading condition are calculated with respect to the baseline values from its standard model.

**Figure 11:**
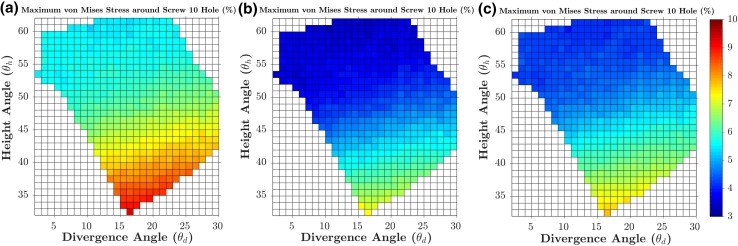
Contour plots showing the percentage changes in the maximum von Mises stress in the bone region 5 mm around screw 10, for each of the 538 feasible height and divergence angle combinations, when subjected to 5 mm (a), 2 mm (b) and 10 mm (c) of varus displacement. Percentage changes for each loading condition are calculated with respect to the baseline values from its standard model.

A fracture gap change Δ*G* of 0.164 mm was calculated from the FE model of the manufacturer’s standard S3 plate subjected to 5 mm varus displacement, which was then used as the baseline to evaluate the 538 models subjected to 5 mm displacement. Figures 8a and 9a show the percentage changes in the Δ*G* and *F*_5_ simulated by the 538 FE models (subjected to 5 mm displacement) in the entire feasible space of the two design parameters (divergence and height angles) with respect to the baseline values. The optimal solution was found at 16° divergence angle and 33° height angle with a 4.686% lower fracture gap change (0.156 mm) and a 5.707% higher *F*_5_ than the baseline model (Figs. 8 and 9). For the standard model subjected to 5 mm displacement, *σ*_mean_ and *σ*_max_ were 1.709 and 12.353 MPa, respectively. These stresses in the 538 models subjected to 5 mm displacement were always higher than those from the standard model; up to 9.259% higher (Figs. 10 and 11). The combination with the highest mean and maximum von Mises stress values was that of 16° divergence angle and 33° height angle.

In general, the results obtained from the 1076 models involving 2 and 10 mm displacement were consistent with those from the aforementioned 5 mm models. For the standard model subjected to 2 mm displacement, Δ*G* and *F*_2_ were 0.066 mm and 21.232 N, respectively. For the standard model subjected to 10 mm displacement, Δ*G* and *F*_10_ were 0.032 mm and 104.381 N. These two standard models were used as baselines for the 1076 models subjected to 2 and 10 mm varus displacement in order to calculate percentage changes. The combination of 16° divergence angle and 33° height angle was still the optimum solution in terms of Δ*G*, *F*_2_ and *F*_10_. For this combination, the fracture gap change was − 4.723% lower, while the *F*_2_ and *F*_10_ values were up to 3.999% higher. Similar to the models subjected to 5 mm displacement, the models of the 2 and 10 mm sets always reported higher mean and maximum Mises stress around screw 10.

Further, the highest von Mises stress values were obtained also with the combination 16° divergence angle and 33° height angle; up to 7.782% higher from the standard model.

**Figure 12:**
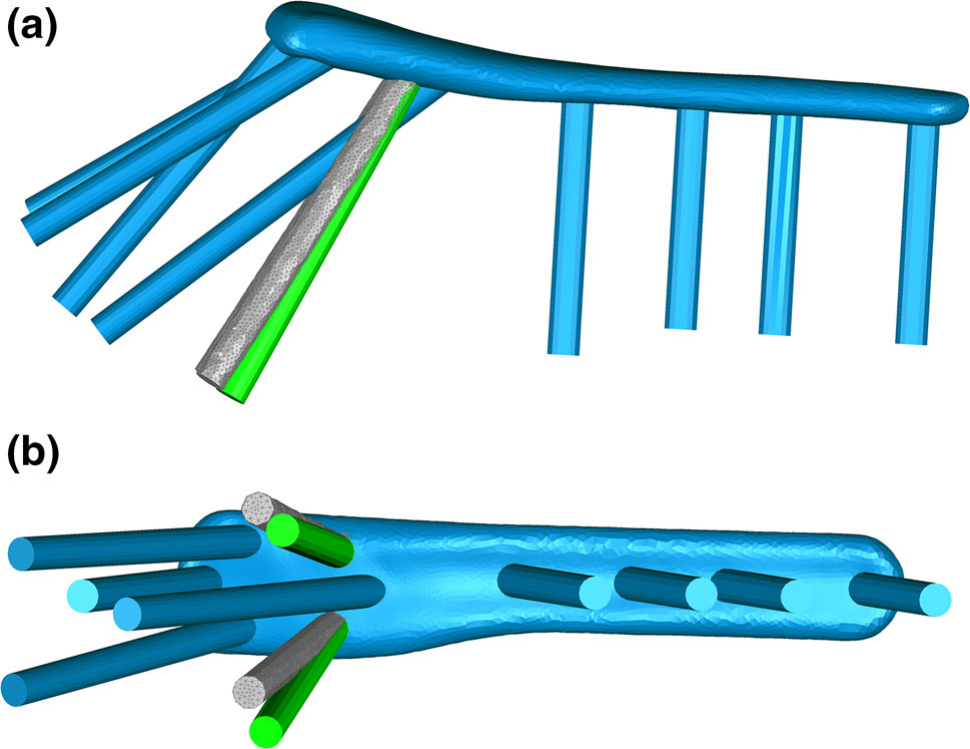
Frontal (a) and sagittal (b) view of the superimposition of the manufacturer’s standard plate (blue, screws 4 and 5 highlighted in green) and the optimal plate design found by the FE-based optimisation (grey).

For all loading conditions (2, 5 and 10 mm), all measured parameters (*F*_2_, *F*_5_, *F*_10_, Δ*G*, mean von Mises stress and maximum von Mises stress) were found to be more sensitive to changes in height angles than divergence angle. In terms of Δ*G* and *F*_5_, the worst solution was at 0° divergence angle and 61° height angle with a 1.926% higher fracture gap change (0.167 mm) and a 1.687% higher *F*_5_ (52.671 N) than the standard values. Δ*G* and *F*_5_ values were more sensitive to changes in height angle than those in the divergence angle. By superimposing the design of the manufacturer’s standard on the optimal design revealed by the parametric optimisation, it can be seen that they share similarities in the orientations of screws 4 and 5 (Fig. 12).

**Figure 13:**
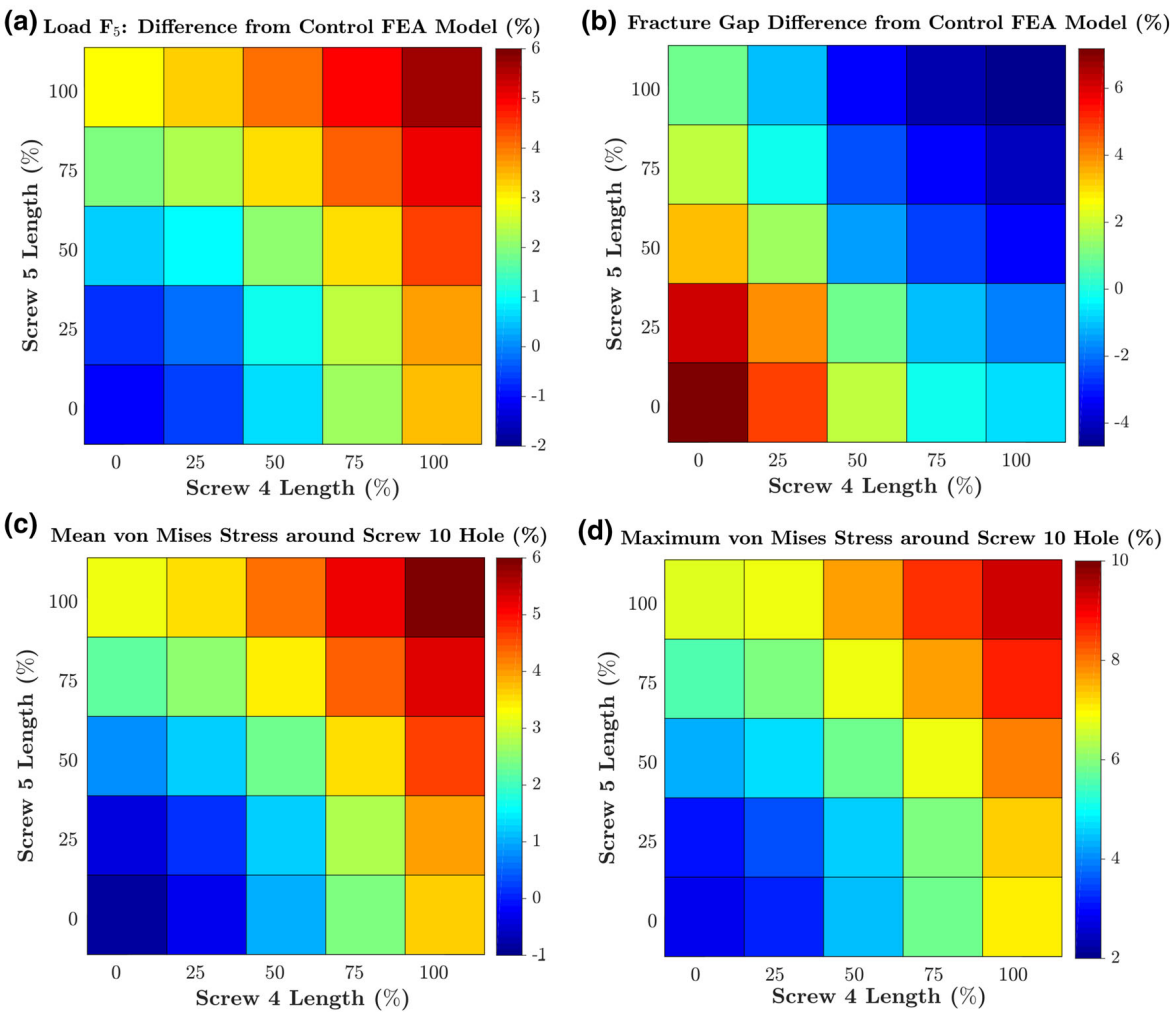
Contour plots showing the percentage changes in the fracture gap change (a), *F*_5_ (b), mean (c) and maximum (d) von Mises stress in the bone region 5 mm around screw 10, for each of the 25 combinations of percentage lengths of screw 4 and 5. Percentage changes for each loading condition are calculated with respect to the baseline values from the 5 mm standard model.

As for the effect of screw length, in general, an increase in the length of either screw 4 or 5 led to an increase in screw in *F*_5_, *σ*_mean_ and *σ*_max_. and reduction in Δ*G* (Fig. 13). Out of these four measurements, Δ*G* was the most sensitive to changes in screw length, as it varied from − 4.686 to 7.180%: a range of approximately 11.9%. The other 3 measurements had ranges between 6 and 7% (*F*_5_, *σ*_mean_ and *σ*_max)_. The worst combination of length, in terms of *F*_5_ and Δ*G* was the removal of both screws 4 and 5. Doing so lead to a construct that had *F*_5_ (1.104% lower) and Δ*G* (7.179% higher) values that were worse than the standard 5 mm model. This highlighted the positive effect of screw length on *F*_5_ and Δ*G* but negative effect on *σ*_mean_ and *σ*_max_.

## Discussion

The current study presents a workflow for computer-aided design optimisation of proximal humerus plates: from humerus CT images to final optimal design. Results show that the proposed FE-based framework is accurate, fast and robust. The FE model was successfully validated against the *in vitro* measurement data for the same bone specimens and under the same loading and boundary conditions. The *F*_5_ value calculated from the FE model was only 0.728% lower than the experimental value and the von Mises stress distribution on the bone-plate construct was comparable to previous studies. This is particularly highlighted by the similarity of the trends obtained for peak loads (*F*_2_, *F*_5_, *F*_10_), Δ*G*, *σ*_mean_ and *σ*_max_ values, despite the differences in displacements applied (2, 5 and 10 mm). The high value of maximum von Mises stress presented in the plate may be attributed to its superior Young’s modulus. The high stresses at the section of the plate spanning the fracture site have been previously reported in several studies, particularly during the debate of optimum plate working length and rigid vs. semi-rigid plates where the minimising of the stresses and strains of this plate section have been discussed.^18^,^20^,^39^ He *et al*. also achieved relatively high von Mises stress in this plate section after applying cantilever load to the shaft in a direction similar to the varus direction.^23^ The high stress concentration found at the last screw hole near the end of the plate during bending is a known phenomenon in literature and may be attributed to the stiffness differences between plate and bone and also the fact that this screw is the support closest to the loading area.^2^,^6^,^12^ Thus, there may be a risk of further fracturing at the peripheral bone-screw junction, especially in patients with osteoporotic bones. Our results show that orientation of screws 4 and 5 could actually increase the local stresses surrounding screw 10.

We significantly increased the speed of the optimisation process by automatically identifying the feasible region of the design parameters. An automated algorithm was developed in Geomagic Wrap, which applied three clinically relevant constraints on the initial search space of 8281 potential FE models to be tested (91 × 91 height and divergence angle combinations). After imposing these constraints, only 538 feasible FE models were found, a 93.503% reduction in search space and thus significant reduction in overall computational time. This filtering of the design space based on clinically pertinent constraints can be applied to design optimisation of other implants of the human body.

Our experiences revealed that manual creation of bone-plate FE model involves several time-consuming tasks: (1) preparation of screws according to specified height and divergence angles; (2) creation of matching bone and screw models (screw hole cutting); (3) selection of surfaces to apply boundary conditions, mesh properties and interactions; (4) successful meshing of the assembly. These tasks demanded human intervention because each of the 538 FE models had a different geometry, meaning that the procedure was not identical for each case and decisions had to be made each time. In this study, we not only automated these four tasks but also the entire process of the FE model creation, including geometrical reconstruction, model assembly and meshing. This led to a 99.896% reduction in the preparation time of each FE model from 8 h to only 30 s. Robustness is one of the key requirements of a successful automation. We generated 538 FE models with a large range of height and divergence angles using the automated process. All the models were found to be soundly constructed when manually checked. When FE analysis was performed on these models, all converged and completed successfully. This was manifested when results similar to the 5 mm optimisation study were achieved in the 1076 FE models involving 2 and 5 mm displacement. It is noteworthy that this automated procedure was achieved for an FE model that involved complex, asymmetrical geometries of the humerus and S3 plate, promising future practical applications to other implant designs and bones.

Varus bending was applied in a cantilever fashion similar to previous biomechanical studies to simulate the supraspinatus pull on the humerus.^10^ Varus direction was specifically selected due to the high complication rate of varus collapse in the clinic.^32^ In both the standard and the optimum S3 plate design, screws 4 and 5 were directed towards the inferomedial region of the humerus. *In vitro* and *in vivo* studies show that mechanically supporting this region is critical for preventing varus collapse. Our biomechanical tests support this finding as out of all the screw zones tested, screws 4 and 5 had the largest effect on the varus bending loads of the bone-plate construct. Further, the optimisation study highlighted the sensitivity of the construct stability (fracture gap change) to inferomedial screws (screws 4 and 5) orientation. The percentage change in fracture gap of the FE models ranged from − 4.686 to + 1.926%. Moreover, by superimposing the design of the standard and the optimum plate, their similarities in the orientation of screws 4 and 5 are visible.

Great care should be taken in the clinic when adjusting the plate’s height since construct stability was found to be sensitive to even small changes in the screws’ height angle. This preference of height angle over divergence angle may be because, unlike the latter, it directly corresponds to screw length along the varus loading direction. Neck-shaft angle, defined as the angle between the anatomic neck and the humeral shaft in the frontal plane, is used in the clinic as a method of determining humeral head’s stability against varus collapse. For healthy humeri, this angle is approximately 135° and more than 100°.^5^,^25^,^35^,^45^ Based on our definition of the height angle, the optimum design had a neck-shaft angle in this range, suggesting a possible relationship between the two angles.

The design optimisation methodology developed in this study can help perform patient-specific optimisation of plates in the clinic. This can be followed by the manufacturing of the optimised design and can theoretically be expanded to the design of implants for other parts of the human body. Custom-made implants for femur, tibia, hip and craniofacial applications have been successfully developed, owing to the modern advances in additive manufacturing and rapid prototyping.^9^,^11^ Even if the manufacturing resources are not available, this design process can be used to conduct a patient-specific optimisation studies on Non-Contact Bridging (NCB) plates to determine optimum screw orientation of each screw. Since the NCB plates have poly-axial locking screws,^46^ this will allow the clinicians to change screws’ orientations to the optimum angles before locking them. This will minimise the need to manufacture a new plate design for each patient.

This study primarily involved the optimisation of two design parameters (*θ*_d_ and *θ*_h_) with a single objective function (Δ*G*), but we also successfully investigated the effect of screw length and aimed to minimise *σ*_mean_ and *σ*_max_. Further studies are required to perform simultaneously optimisation of multiple design parameters, such as plate’s geometry, locking screws’ geometry, number and position. A more extensive multi-objective optimisation may be also required, for example, to determine the design with minimum fracture gap change and the von Mises stresses. To achieve that, alternative numerical optimisation algorithms, such as genetic algorithm, may be needed.

As for the limitations of this study, first, the biomechanical tests and the FE models in this study, only involved testing of synthetic humeri. Tests on cadaveric humeri are required to develop FE models with regional differences in cortical and cancellous microstructures, allowing a more accurate calculation of stresses and loads. This may also affect the values of *σ*_mean_ and *σ*_max_. Moreover, we modelled perfect locking of the screws by merging them to the plate and modelled the ideal bone-screw purchase by tying the screws to the bone, as described by Zhang *et al*.^47^ This was because the pull-out of screws was not observed in our *in vitro* tests. However, post-operative screw pull-out has been reported in the clinic and thus the screws may need to be modelled separately and with frictional surface properties, especially if the loading conditions are changed.^7^ Finally, we only optimised the plate design for varus bending and this may have compromised the stability in other directions. Simulation of more complex *in vivo* movements such as glenohumeral abduction needs to be performed, for which the application of cyclic loading and modelling of the bones, tendons and musculature surrounding the humerus may be needed.

In conclusion, the FE-based design optimisation framework presented in this study, is accurate, fast and robust, thanks to its experimental validation against *in vitro* biomechanical tests, the automated explicit identification of the feasible design space and the complete automation of the FE model creation process. To test the robustness of our technique, we conducted additional studies that investigated the effect of factors such as loading conditions and screw length, on varus stability and these models demonstrated consistency in optimisation results. The model yielded F_5_ values that were only 0.728% less than *in vitro* biomechanical tests. The identification of the feasible space based on clinically relevant constraints reduced the search space by 93.503%, and the automated model construction process reduced the preparation time of each FE model by 99.896%. The *in vitro* biomechanical tests and the FE simulation results suggested that the varus bending stiffness of the bone-plate construct is more dependent on the screw 4 and 5 than any other screw zones. The optimum height angle obtained was found to be similar to the neck-shaft angle of a healthy subject and demands further investigation. Findings of this study reveal valuable information for plate-based treatment of proximal humerus fractures while the methodological novelties of this study can be implemented for the design optimisation of other implants of the human body.
